# Glucocorticoid receptor activation stimulates the sodium-chloride cotransporter and influences the diurnal rhythm of its phosphorylation

**DOI:** 10.1152/ajprenal.00372.2019

**Published:** 2019-10-07

**Authors:** Jessica Ruth Ivy, Natalie K. Jones, Hannah M. Costello, Morag K. Mansley, Theresa S. Peltz, Peter W. Flatman, Matthew A. Bailey

**Affiliations:** British Heart Foundation Centre for Cardiovascular Science, Edinburgh Medical School, The University of Edinburgh, United Kingdom

**Keywords:** blood pressure, circadian rhythm, distal convoluted tubule, glucocorticoid, sodium-chloride cotransporter

## Abstract

The sodium-chloride cotransporter (NCC) in the distal convoluted tubule contributes importantly to sodium balance and blood pressure (BP) regulation. NCC phosphorylation determines transport activity and has a diurnal rhythm influenced by glucocorticoids. Disturbing this rhythm induces “nondipping” BP, an abnormality that increases cardiovascular risk. The receptor through which glucocorticoids regulate NCC is not known. In this study, we found that acute administration of corticosterone to male C57BL6 mice doubled NCC phosphorylation without affecting total NCC abundance in both adrenalectomized and adrenal-intact mice. Corticosterone also increased the whole kidney expression of canonical clock genes: period circadian protein homolog 1 (*Per1*), *Per2*, cryptochrome 1, and aryl hydrocarbon receptor nuclear translocator-like protein 1. In adrenal-intact mice, chronic blockade of glucocorticoid receptor (GR) with RU486 did not change total NCC but prevented corticosterone-induced NCC phosphorylation and activation of clock genes. Blockade of mineralocorticoid receptor (MR) with spironolactone reduced the total pool of NCC but did not affect stimulation by corticosterone. The diurnal rhythm of NCC phosphorylation, measured at 6-h intervals, was blunted by chronic GR blockade, and a similar dampening of diurnal variation was seen in GR heterozygous null mice. These effects on NCC phosphorylation did not reflect altered rhythmicity of plasma corticosterone or serum and glucocorticoid-induced kinase 1 activity. Both mineralocorticoids and glucocorticoids emerge as regulators of NCC, acting via distinct receptor pathways. MR activation provides maintenance of the NCC protein pool; GR activation dynamically regulates NCC phosphorylation and establishes the diurnal rhythm of NCC activity. This study has implications for circadian BP homeostasis, particularly in individuals with abnormal glucocorticoid signaling as is found in chronic stress and corticosteroid therapy.

## INTRODUCTION

The main sodium entry pathway in the distal convoluted tubule (DCT) is the thiazide-sensitive sodium-chloride cotransporter (NCC). NCC-mediated salt reabsorption contributes importantly to blood pressure (BP) regulation: null mutations in the encoding gene, *SLC12A3*, cause Gitelman’s syndrome, characterized by urinary salt wasting and low BP; “gain-of-function” in NCC, arising from mutations in key regulatory kinases and ubiquitin ligases, causes the hypertensive disorder of pseudohypoaldosteronism type II (25). NCC activity is modulated by multiple hormonal systems (41) and also directly by plasma potassium concentration (37, 47). In addition to control of NCC abundance per se, hormones and other factors engage a regulatory cascade of serine/threonine kinases, including with-no-lysine kinase (WNK)1 and WNK4, STE20/SPS1-related proline/alanine-rich kinase, and oxidative stress-response kinase 1 (2). These kinases influence phosphorylation at conserved residues in the NH_2_-terminus, influencing trafficking in the apical membrane of the DCT and establishing overall NCC activity in vivo. In the mouse, three critical residues are Thr^53^, Thr^58^, and Ser^71^, with Thr^53^ phosphorylation, measured in the current study, increasing membrane retention and activity of NCC (40, 43).

Circadian transcription factors influence renal function (11) and BP (12). The DCT expresses the canonical circadian transcription factors period circadian protein homolog 1 (*Per1*), *Per2*, aryl hydrocarbon receptor nuclear translocator-like protein (*Arntl or Bmal1*), *Clock*, and cryptochrome 1/2 (*Cry1* and *Cry2*) (54). In a DCT cell line, *Per1* transcriptionally regulates WNKs and phosphorylation of NCC (39). In humans (8) and rodents (19, 49), phosphorylated (p)NCC has a marked diurnal variation, being higher in the active phase and lower in the inactive, or sleep, phase of the 24-h cycle. Rhythmic physiology is synchronized to the day/night cycle by the master clock in the suprachiasmatic nucleus (SCN) of the hypothalamus, which itself is entrained by the photoperiod. Glucocorticoids (cortisol in humans and corticosterone in rodents) entrain peripheral clocks, including those in the kidney, to the rhythm established centrally by the SCN (4, 33). We recently found that abolishing diurnal variation in circulating glucocorticoid by adrenalectomy or chronic corticosterone infusion flattened the diurnal variation in pNCC and induced nondipping BP (19). Nondipping, aka nocturnal, hypertension in humans increases cardiovascular risk (45) and precedes and predicts nephropathy in diabetes (23). In our mouse study, thiazide partially restored normal diurnal BP variation in corticosterone-treated mice (19).

The receptor through which glucocorticoids regulate NCC activity is not known. Glucocorticoids have approximately equal affinity for the glucocorticoid receptor (GR) and the mineralocorticoid receptor (MR), and DCT cells express both receptors (1). The distal nephron is conventionally considered aldosterone sensitive because of high expression of 11β-hydroxysteroid dehydrogenase type 2 (11βHSD2), which metabolizes and inactivates cortisol/corticosterone. However, we (17) and others (7, 28, 34) have shown that the DCT expresses weak/undetectable levels of 11βHSD2, which may be restricted to the short DCT2 segment. We therefore proposed that glucocorticoids can activate MR to increase pNCC. To test this hypothesis, we chronically treated mice with either spironolactone, a MR antagonist, or RU486, a GR antagonist, and assessed the effect of acute administration of corticosterone on pNCC. In a separate experiment, the effect of RU486 on the diurnal variation of NCC phosphorylation was measured.

## METHODS

### 

#### Animals.

Male C57BL6J/Ola mice (Harlan) were used for *experiments 1*–*3*. Male mice haploinsufficient for the GR (GR^βgeo/+^, as described in Refs. 18 and 27) and their wild-type littermates (GR^+/+^) were used for *experiment 4*. Mice were between 2 and 4 mo of age and were acclimatized to a 12:12-h light-dark cycle for at least 2 wk before experiments and given free access to water and standard chow (maintenance diet 1; Special Diet Services, Essex, UK). The lights were turned on at 7:00 AM local time, and this was defined as *Zeitgeber* *time 0* (ZT0); lights were turned off at 7:00 PM (ZT12). All experiments were performed in accordance with the United Kingdom’s Animals (Scientific Procedures) Act of 1986 and under the authority of a project license issued by the United Kingdom Home Office following approval by the University’s Animal Welfare & Ethical Review Board.

#### Tissue collection.

Mice were terminated by cervical dislocation within 1 min of removal from the holding room. A laparotomy was performed, and the kidneys were removed, decapsulated, snap frozen on dry ice, and stored at −80°C for Western blot analysis and quantitative PCR (qPCR) analysis. A terminal plasma sample was taken via the abdominal vena cava and used to measure plasma corticosterone in some experiments.

#### Plasma corticosterone.

Plasma corticosterone was measured using a commercially available corticosterone EIA kit (Enzo Life Sciences). All reagents were provided in the kit. Plasma corticosterone was extracted from plasma samples by incubating 10 μL of sample with 10 μL of steroid displacement reagent [SDR; diluted 1:100 with assay buffer 15 (AB15)] for 5 min at room temperature (RT) in sterile glass tubes. AB15 (280 μL) was then added along with 3 mL ethyl acetate (Sigma-Aldrich), and the glass tube was vortexed thoroughly, incubated (RT, 2 min), and vortexed again. The clear organic upper layer was aspirated to a new glass tube. The samples were desiccated under nitrogen at 60°C. Standards and samples were prepared in duplicate and assayed according to the manufacturer’s instructions. Absorbance measurements (405 nm and corrected with 580 nm) were plotted against the known standard values using GraphPad software, the *r*^2^ value for the sigmoidal curve produced was >0.98, and the concentrations of the unknown samples were interpolated on the line.

#### Plasma electrolyte measurements.

Plasma potassium and sodium were measured in two parallel cohorts of animals: first, in mice acutely injected with corticosterone (*n* = 10) or vehicle (*n* = 9) and, second, in mice treated for 5 days with RU486 (*n* = 3) or vehicle (*n* = 4). To avoid hemolysis, blood was drawn from an arterial line under general anesthesia, induced by intraperitoneal injection of 100 mg/kg thiopentalbarbitol. The carotid artery was cannulated using PE-10 tubing containing 20 U/L porcine heparin saline (Sigma-Aldrich). After the heparin saline had been cleared from the tubing, an ~500-μL sample of arterial blood was collected in lithium/heparin-coated tubes, and plasma was separated by centrifugation. Plasma electrolytes were measured using an electrolyte analyzer (Roche).

#### Immunoblot analysis.

Western blots were performed on homogenized whole kidney samples as we have described (17) using primary antibodies against NCC (Chemicon) and pThr^53^-NCC. We have previously reported that pThr^53^-NCC abundance had a marked diurnal variation (19). Images were developed by electrochemiluminescence and quantified by densitometry using ImageJ, as described (26). Coomassie blue staining was adapted for use as a loading control to normalize the immunoblot results for the amount of protein loaded on the gel. A dose curve indicated that densitometry of the Coomassie blue staining was linear in the range of 10–40 μg protein. The bottom of the gel, just above the 55-kDa mark, was trimmed and incubated in Coomassie blue solution (RT, 3–4 h) and then destaining solution overnight. For occasions where two or more Western blots were required to run the samples, these were run contemporaneously using the same conditions, and a reference load was prepared from a pool of homogenate run on each gel to normalize the data to account for interrun differences.

#### Quantitative PCR.

RNA was extracted from half kidneys using RNeasy spin columns (QIAGEN), and the RNA concentration was quantified using a Nanodrop 1000 spectrophotometer. RNA concentrations were 200–700 ng/μL. All samples taken forward for reverse transcription had ratios of absorbance at 260 to 280 nm of 1.7–2.1. The absorbance ratios of 260 to 230 nm were usually in the range of 1.8–2.2. Reverse transcription was carried out on 500 ng RNA using the AB High Capacity RT Kit (Life Technologies). Included in each RT-PCR round were a no-template control and a no-reverse transcriptase control. Resultant cDNA samples were stored at −20°C before dilution for qRT-PCR. Assays for qPCR were designed using the Roche Universal Probe library so that the amplicon spanned an intron excluding the first or last intron to avoid targeting any single-nucleotide polymorphisms. Primers were then screened through the Integrated DNA technologies oligo analyzer tool (http://www.idtdna.com/calc/analyzer) to identify those with any hairpins, self-dimers, and heterodimers. All selected primers were purchased from Eurofins Scientific. Each assay was quality controlled and rejected if the standard curve error exceeded 0.05 (i.e., the mean squared error of the data points making up the standard curve) or the efficiency was outside the range of 1.7–2.1. PCR products were also examined with loading dye (DNA Gel Loading Dye, 6X; Life Technologies) on a 4% Tris-borate-EDTA agarose gel with 0.05% ethidium bromide to ensure the amplicons conformed to the expected lengths. Primers and probe combinations are given in Table 1. TATA box-binding protein (TBP), hypoxanthine-guanine phosphoribosyltransferase (HPRT), and 18S rRNA were used as endogenous control genes.

**Table 1.** Quantitative RT-PCR assay details

Gene	Description (Protein Abbreviation)	Forward Primer (5′ to 3′)	Reverse Primer (5′ to 3′)	Probe, bp
*Rn18s*	18S rRNA	ctcaacacgggaaacctcac	cgctccaccaactaagaacg	77
*Tbp*	TATA box-binding protein (TBP)	gggagaatcatggaccagaa	gatgggaattccaggagtca	97
*Hprt1*	Hypoxanthine-guanine phosphoribosyltransferase (HPRT)	cctcctcagaccgcttttt	aacctggttcatcatcgctaa	95
*Per1*	Period circadian protein homolog 1 (PER1)	gcttcgtggacttgacacct	tgctttagatcggcagtggt	71
*Per2*	Period circadian protein homolog 2 (PER2)	gcttcgtggacttgacacct	tgctttagatcggcagtggt	5
*Clock-201*	Circadian locomotor output cycles protein kaput (CLOCK)	ccagtcagttggtccatcatt	tggctcctaactgagctgaaa	76
*Cry1*	Cryptochrome 1 (CRY1)	ggcagagcagtaactgatacga	tgactttcccaccaacttca	52
*Cry2*	Cryptochrome 2 (CRY2)	ggagcatcagcaacacagg	ccgcttggtcagttcttcac	11
*Arnt1 var1*	Aryl hydrocarbon receptor nuclear translocator-like protein 1 (BMAL1)	gaatacattgtctcaaccaacactg	ttagctgcgggaaggttg	97
*Nr1d1*	Nuclear receptor subfamily 1 group D member 1 (Rev-erbα)	cgaccctggactccaataac	tgccattggagctgtcact	52
*Nr1d2*	Nuclear receptor subfamily 1 group D member 2 (Rev-erbβ)	stggagctgaacgcagga	tcagaaccctcactgtgacaa	16
*Sgk1*	Serum- and glucocorticoid-induced kinase 1 (SGK1)	gattgccagcaacacctatg	ttgatttgttgagagggacttg	91
*Tsc22d3 v2*	Glucocorticoid-induced leucine zipper protein (GILZ)	tccgttaaactggataacagtgc	tggttcttcacgaggtccat	49
*Nr3c2*	Mineralocorticoid receptor (MR)	caaaagagccgtggaagg	tttctccgaatcttatcaataatgc	11
*Nr3c1*	Glucocorticoid receptor (GR)	tgacgtgtggaagctgtaaagt	catttcttccagcacaaaggt	56
*Hsd11b2*	11β-Hydroxysteroid dehydrogenase type 2 (11βHSD2)	cactcgaggggacgtattgt	gcaggggtatggcatgtct	26
*Slc12a3*	Sodium-chloride cotransporter (NCC)	cctccatcaccaactcacct	ccgcccacttgctgtagta	12
*Wnk4*	With-no-lysine kinase 4 (WNK4)	tccgatttgatctggatgg	gggcaggatgaactcattgta	26

Assays were designed using the Roche Universal Probe library, and assays were run using Roche Universal Probe library probes.

#### qPCR Quantification.

Quantitation was achieved using the automated LightCycler 480 software, which identifies the threshold cycle (C_T_) value for each well by finding the maximum value from a plot of second derivative of fluorescence versus time. Each triplicate value was analyzed, and replicates were excluded if the C_T_ SD was >0.5. Results were accepted if the standard curve was adequate, as assessed by efficiency within the range of 1.7–2.1 and error <0.05 (i.e., the mean squared error of the data points making up the standard curve). Endogenous control genes were used to normalize the experimental gene values. A panel of three endogenous control genes was used, including TBP, HPRT, and 18S rRNA (15, 32, 46). The expression of these control genes did not differ across any of the experimental groups, and C_T_ values were ~25–33, 22–28, and 7–15 for TBP, HPRT, and 18S, respectively. Test gene C_T_ values ranged from 22 to 37. The level of endogenous control genes was normalized such that the control sample was equal to one (so as not to give greater weight to any one of the control genes), and the mean of all three control genes was taken. The experimental gene expression was then calculated as the mean level normalized to the mean of the control genes and log10 transformed to normalize the spread of data.

#### Experiment 1: acute corticosterone administration in adrenalectomized mice.

Bilateral adrenalectomy was performed under isofluorane anesthesia and buprenorphine (0.1 mg/kg sc Vetergesic) analgesia. After recovery, adrenalectomized (ADX) and control mice were individually housed and given ad libitum access to 0.9% saline and tap water. An “adrenal-intact” group (*n* = 8) was anesthetized but not surgically manipulated. At *postoperative day 9*, ADX animals were injected subcutaneously at ZT1 with either vehicle (2% DMSO in 0.9% sterile injectable saline, *n* = 8) or 6 mg/kg corticosterone (*n* = 10). Mice were terminated at ZT5, and kidneys were taken. Adrenal-intact animals (*n* = 10) were not injected but terminated at the same time as ADX animals.

#### Experiment 2: acute corticosterone treatment with chronic MR or GR blockade in adrenal-intact mice.

Elastomer pellets were used to encapsulate spironolactone (MR antagonist) or RU486 (GR antagonist) for slow release as previously described (3). Pellets contained ~30 mg of drug, and, in vitro, the release rate was ~3 mg·kg^−1^·day^−1^. In each mouse, two pellets were implanted subcutaneously under isofluorane anesthesia and buprenorphine analgesia (0.1 mg/kg sc Vetergesic). Groups were as follows: spironolactone, RU-486, or blank. After 5 days, mice from each of these three treatment groups were then injected at ZT0 with either vehicle or 6 mg/kg corticosterone (sc). Mice were terminated at ZT4, and kidneys were taken for analysis.

#### Experiment 3: RU486 and the diurnal variation of NCC phosphorylation.

Mice were treated for 5 days with 60 mg RU486 or no-drug pellet and terminated for tissue collection (as above) at ZT0, ZT6, ZT12, and ZT18. Mice were pair housed with one treated with RU-486 and the other with vehicle and terminated contemporaneously for tissue collection (as above) at ZT6 and ZT18.

#### Experiment 4: GR heterozygotes and diurnal variation of NCC phosphorylation.

GR^βgeo/+^ and GR^+/+^ mice were pair housed and terminated in pairs together for tissue collection (as above) at ZT6 and ZT18.

#### Statistical analysis.

Data are presented as individual points with group means ± SD. The number of biological replicates (*n*) for each experimental group is given in the figures along with the test used for statistical analysis. Data were analyzed by *t*-tests or, where there was more than one dependent factor, two-way ANOVA with post hoc Sidak’s tests.

## RESULTS

### 

#### Corticosterone increases NCC phosphorylation in ADX rats.

**Fig. 1.**
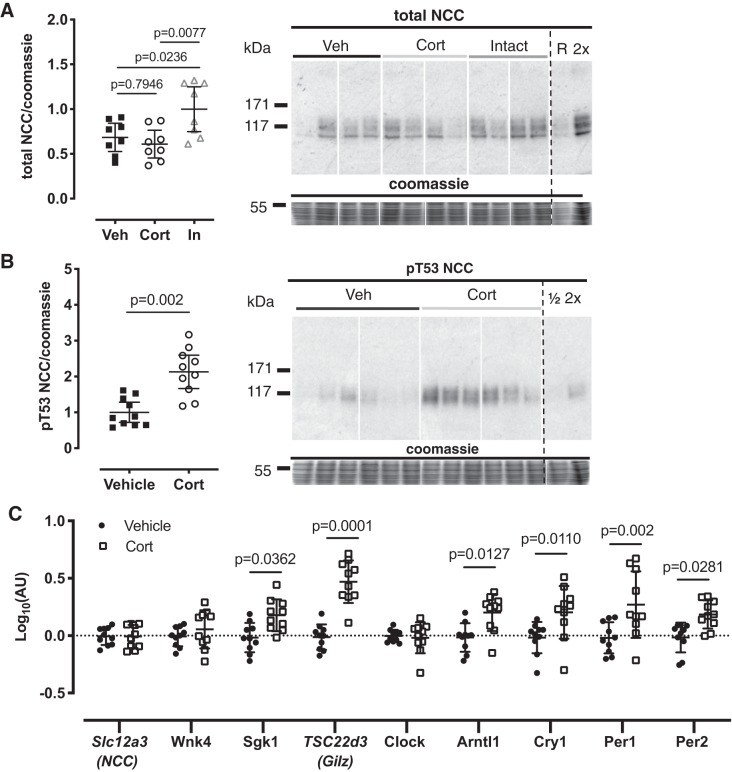
Adrenalectomy reduces total levels of the sodium-chloride cotransporter (NCC, *A*). In adrenalectomized animals, acute corticosterone injection induced an upregulation of NCC phosphorylation at Thr^53^ (*B*), accompanied by changes in gene expression (*C*). C57BL6 mice were adrenalectomized [vehicle (Veh)/corticosterone (Cort)] or undisturbed (In), and all were maintained on 0.9% saline in place of drinking water. Mice then received an acute intraperitoneal injection of vehicle (Veh/Intact) or corticosterone, and kidneys were taken 6 h later for Western blot analysis. All samples were run on the same blots or evenly distributed across blots and normalized to a reference lane. All rearrangements of blots are for clarity. Signal densities for immunoblots were normalized to Coomassie signal densities and to the reference lane (R) and were analyzed by one-way ANOVA, followed by post hoc Tukey’s tests (*n* = 8 biological replicates, *A*), *t*-tests (*n* = 10, *B*), or two-way ANOVA followed by post hoc Sidak’s tests (*n* = 10, *C*). Data are means ± SD. See Table 1 for gene descriptions and protein names.

Following bilateral adrenalectomy (5 days), total NCC abundance was significantly reduced in ADX mice compared with adrenal-intact mice (Fig. 1*A*). ADX mice were injected intraperitoneally with a bolus of either corticosterone or vehicle at ZT0. Kidneys were taken at ZT4, and the abundance of pT53-NCC, measured by Western blot, was approximately twofold higher in corticosterone-injected mice compared with vehicle controls (Fig. 1*B*). There was no difference in mRNA abundance for the NCC encoding gene, *Slc12a3* (Fig. 1*C*). Acute corticosterone did, however, increase the whole kidney mRNA expression of serum- and glucocorticoid-induced kinase 1 (*Sgk1*) and the gene encoding glucocorticoid-induced leucine zipper protein (GILZ, Fig. 1*C*). The expression of clock genes *Arntl1*, *Cry1*, *Per1*, and *Per2* was significantly increased by acute administration of corticosterone to ADX mice (Fig. 1*C*). Plasma potassium concentration, an important regulator of NCC phosphorylation, was measured in parallel groups of mice and found to be lower in corticosterone-treated mice (*P* = 0.06, Table 2).

**Table 2.** Plasma sodium and potassium from C57BL6 mice treated acutely with corticosterone (6 mg/kg sc) or chronically for 5 days with RU486 versus control

Treatment Group	Plasma Sodium, mmol/L	*P* Value (Plasma Sodium)	Plasma Potassium, mmol/L	*P* Value (Plasma Potassium)	*n*
Acute vehicle	143.5 ± 2.2	0.87	4.05 ± 0.3	0.06	10
Acute Corticosterone	143.3 ± 2.2		3.81 ± 0.2		9
Control	146.3 ± 2.6	0.34	4.25 ± 0.3	0.10	4
Chronic RU486	148.3 ± 2.5		3.80 ± 0.2		3

Data are means ± SD and were analyzed by Student’s *t*-tests; *n*, no. of biological replicates.

#### Chronic GR antagonism prevents acute phosphorylation of NCC by corticosterone.

**Fig. 2.**
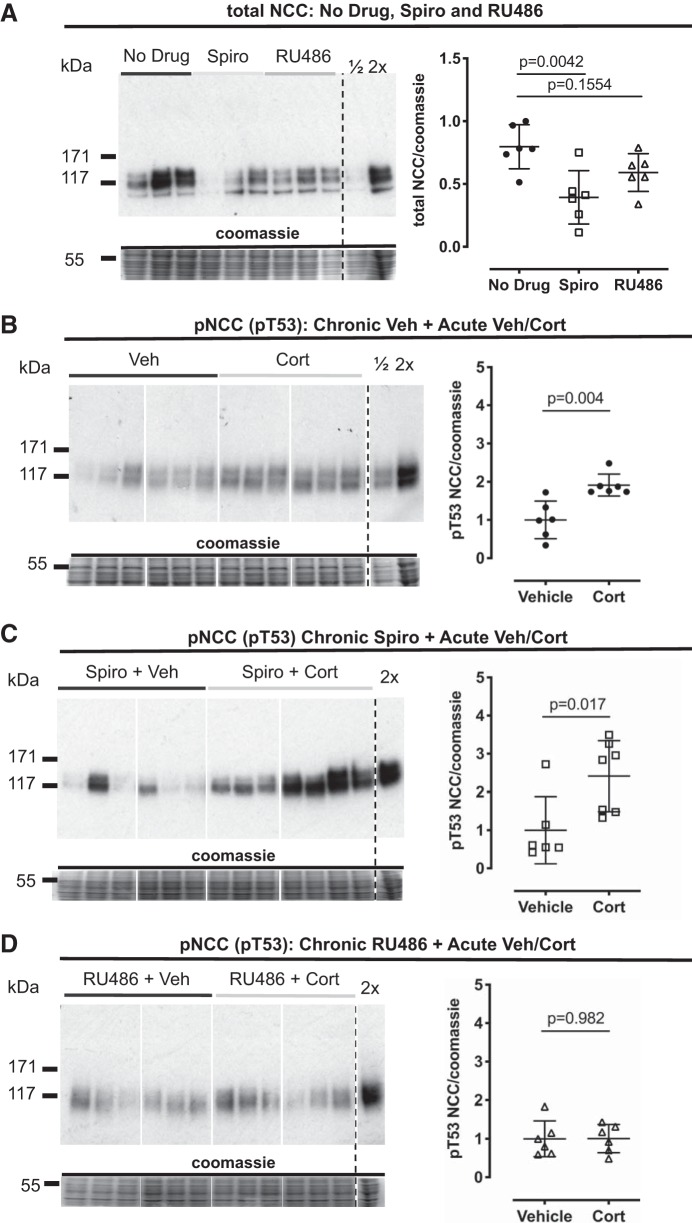
In adrenal-intact mice, sodium-chloride cotransporter (NCC) total protein is reduced by mineralocorticoid receptor (MR) but not glucocorticoid receptor (GR) antagonism; however, GR antagonism prevents corticosterone (Cort)-induced NCC phosphorylation at Thr^53^ (T53). *A*: NCC protein was measured in whole kidney homogenate taken from mice treated for 5 days with a slow-release Silastic pellet containing spironolactone (Spiro, 50 mg), RU486 (60 mg), and in controls (no-drug pellet). NCC phosphorylation at threonine (pT53) was measured in whole kidney homogenate taken from untreated mice (no drug, *B*), 5-day spironolactone-treated (50 mg, *C*), and 5-day RU486-treated (*D*) mice, 4 h following acute corticosterone administration. Samples were run on the same blots (*B*–*D*) or evenly distributed across blots and normalized to a reference lane (*A*). All rearrangements of blots are for clarity. Signal densities for immunoblots were normalized to Coomassie signal densities. Veh, vehicle. Data are means ± SD and were analyzed by one-way ANOVA (*A*) and Student’s *t*-tests (*B*–*D*), *n* = 6 biological replicates.

To resolve the receptor pathways through which corticosterone activated NCC, we used adrenal-intact mice pretreated with either spironolactone (MR antagonist) or RU486 (GR antagonist). Control mice were implanted with a no-drug pellet. Chronic MR blockade by spironolactone significantly reduced total NCC abundance compared with the no-drug group, but total NCC was unchanged in RU486-treated animals (Fig. 2*A*). Acute injection of corticosterone at ZT0, the nadir of the endogenous corticosterone rhythm, significantly increased pT53-NCC in the no-drug controls (Fig. 2*B*) and in the mice pretreated with spironolactone (Fig. 2*C*). In contrast, acute corticosterone did not increase pT53-NCC in mice chronically treated with RU486 (Fig. 2*D*). Notably, plasma potassium tended to be lower in RU486-treated mice (Table 2).

**Fig. 3.**
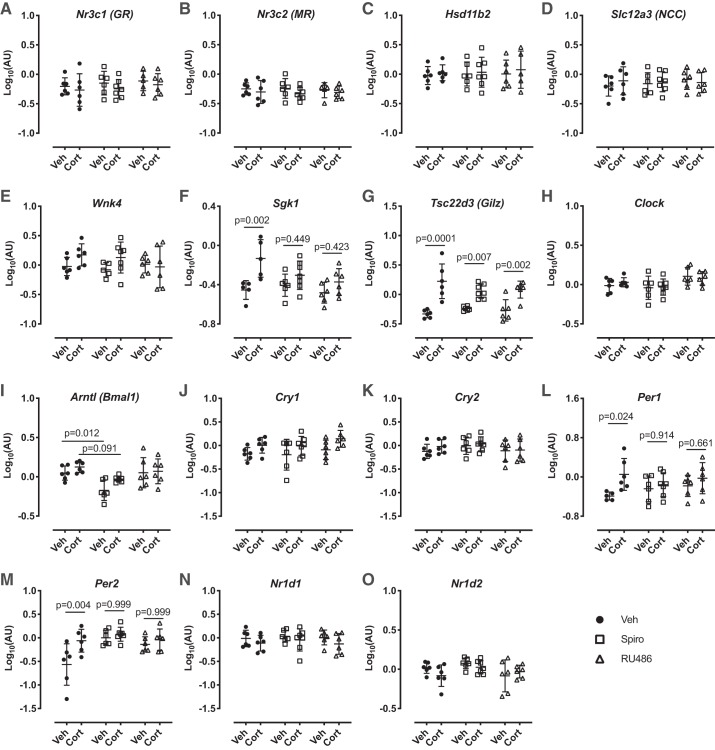
Gene expression in C57BL6 mouse kidneys in response to 5-day vehicle (black circles), chronic spironolactone (Spiro, 50 mg, white squares), or chronic RU486 (60 mg, white triangles) treatment and following acute corticosterone (Cort, 6 mg/kg sc) or vehicle (2% DMSO, Veh) treatment. Data are means ± SD and were analyzed by two-way ANOVA followed by post hoc Sidak’s tests, *n* = 5–7 biological replicates. GR, glucocorticoid receptor; MR, mineralocorticoid receptor; HSD11b2, 11β-hydroxysteroid dehydrogenase type 2; Slc12a3, sodium-chloride cotransporter; NCC, sodium-chloride cotransporter; Wnk4, with-no-lysine kinase 4; Sgk1, serum and glucocorticoid-induced kinase 1; Tsc22d3 (Gilz), glucocorticoid-induced leucine zipper protein; Clock, circadian locomotor output cycles protein kaput; Arntl, aryl hydrocarbon receptor nuclear translocator-like protein 1; Cry1, cryptochrome 1; Cry2, cryptochrome 2; Per1, period circadian protein homolog 1; Per2, period circadian protein homolog 2; Nrld1, nuclear receptor subfamily 1 group D member 1; Nrld2, nuclear receptor subfamily 1 group D member 2.

**Fig. 4.**
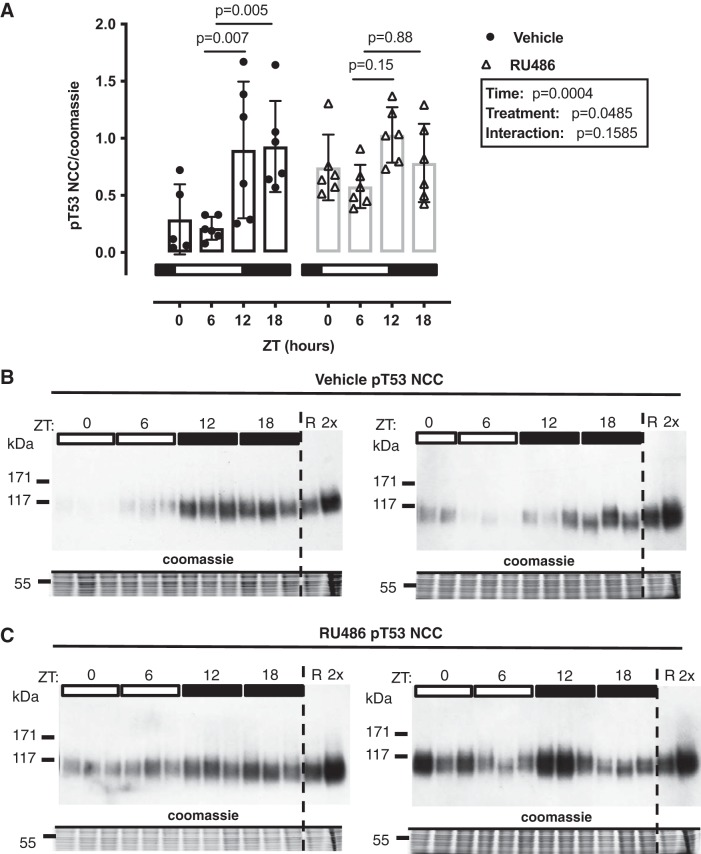
Diurnal change in sodium-chloride cotransporter (NCC) phosphorylation at Thr^53^ (T53) is abrogated by chronic RU-486 treatment. Male C57BL6 mice were treated for 5 days with RU486 slow-release pellets or vehicle and terminated in pairs every 6 h starting at *Zeitgeber* *time* (ZT) *0* (lights on, local time 7:00 AM). Summary data are presented for Western blots run in parallel (*A*), and Western blots are presented in *B* and *C*. R, reference pool of kidney homogenate loaded on all gels; 2×, 2× loading control to test linearity. Data are means ± SD and analyzed by two-way ANOVA with post hoc Sidak’s tests comparing data within the same genotype to the trough levels at ZT6, *n* = 5–6 biological replicates.

We again profiled whole kidney mRNA expression of a panel of genes known to influence NCC expression. Neither chronic spironolactone nor RU486 affected the expression of *Nr3c1* (encoding GR; Fig. 3*A*), *Nr3c2* (encoding MR; Fig. 4*B*), *Hsd11b2* (encoding 11βHSD2; Fig. 4*C*), or *Slc12a3* (encoding NCC; Fig. 3*D*). Acute administration of corticosterone did not change mRNA abundance of these genes. We again found that acute corticosterone significantly increased the expression of S*gk1* (Fig. 3*F*) and *Tsc22d3* (encoding GILZ; Fig. 3*G*). The effect on *Sgk1* was significantly blunted both by MR and GR antagonism; the stimulatory effect on *Tsc22d3* persisted during chronic MR and GR blockade.

#### Chronic GR antagonism blunts the diurnal variation in NCC phosphorylation.

**Fig. 5.**
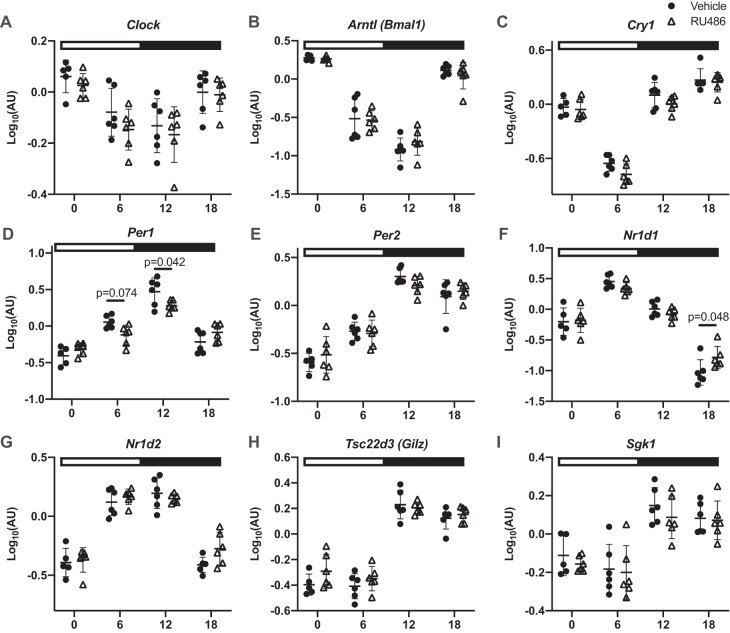
Diurnal variation in gene expression in mice treated with chronic RU486 (white triangles) or vehicle (black circles). Male C57BL6 mice were treated for 5 days with RU486 slow-release pellets or vehicle and terminated in pairs every 6 h starting at *Zeitgeber* *time* (ZT) *0* (lights on, local time 7:00 AM). Kidneys were taken for quantitative RT-PCR. Clock, circadian locomotor output cycles protein kaput; Arntl, aryl hydrocarbon receptor nuclear translocator-like protein 1; Cry1, cryptochrome 1; Cry2, cryptochrome 2; Per1, period circadian protein homolog 1; Per2, period circadian protein homolog 2; Nrld1, nuclear receptor subfamily 1 group D member 1; Nrld2, nuclear receptor subfamily 1 group D member 2; Tsc22d3 (Gilz), glucocorticoid-induced leucine zipper protein; Sgk1, serum- and glucocorticoid-induced kinase 1. Data are means ± SD and were analyzed by two-way ANOVA followed by post hoc Sidak’s tests, *n* = 5–6 biological replicates. See Table 1 for gene descriptions and protein names.

**Fig. 6.**
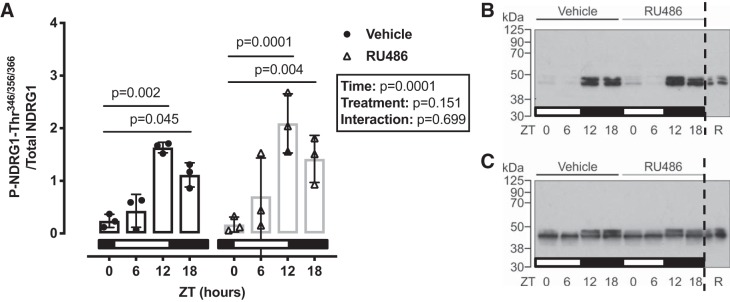
*A*: serum- and glucocorticoid-induced kinase 1activity [estimated by *n*-myc downstream regulated kinase 1 (NDRG1) phosphorylation (P) at Thr^349,356,366^] has a diurnal rhythm that is unaffected by RU486 treatment. Representative blots show P-NDRG1-Thr (*B*) and total NDRG1 (*C*). Western blot analysis were run in parallel. R, reference pool of kidney homogenate loaded on all gels. Data are means ± SD and were analyzed by two-way ANOVA followed by post hoc Sidak’s tests, *n* = 3 biological replicates.

pT53-NCC has a diurnal rhythm that is influenced by plasma corticosterone (19). Therefore, we next assessed the effect of RU486 pretreatment on pT53-NCC measured at 6-h intervals through the 24-h cycle. In untreated mice, implanted with a no-drug pellet, pT53-NCC showed a marked diurnal variation and was higher at ZT12 and ZT18 than at ZT0 and ZT (Fig. 4, *A* and *B*). In RU486-treated mice, pT53-NCC was higher at ZT0 and ZT6 than in no-drug controls; this significantly blunted the diurnal variation in pT53-NCC abundance (Fig. 4, *A* and *C*). Surprisingly, only Per1 and Nr1d1 expression was altered by chronic RU486 treatment; the rhythm of other clock genes remained intact (Fig. 5). We also assessed the activity of SGK1 at each time point, using the phosphorylation of *n*-myc downstream regulated kinase 1 (NDRG1) as a surrogate. NDRG1 is phosphorylated by SGK1 but not by other closely related kinases and therefore can be used as a measure of endogenous SGK1 activity (24). SGK1 activity displayed a marked diurnal variation, being higher in subjective night; this was not affected by chronic RU486 treatment (Fig. 6).

**Fig. 7.**
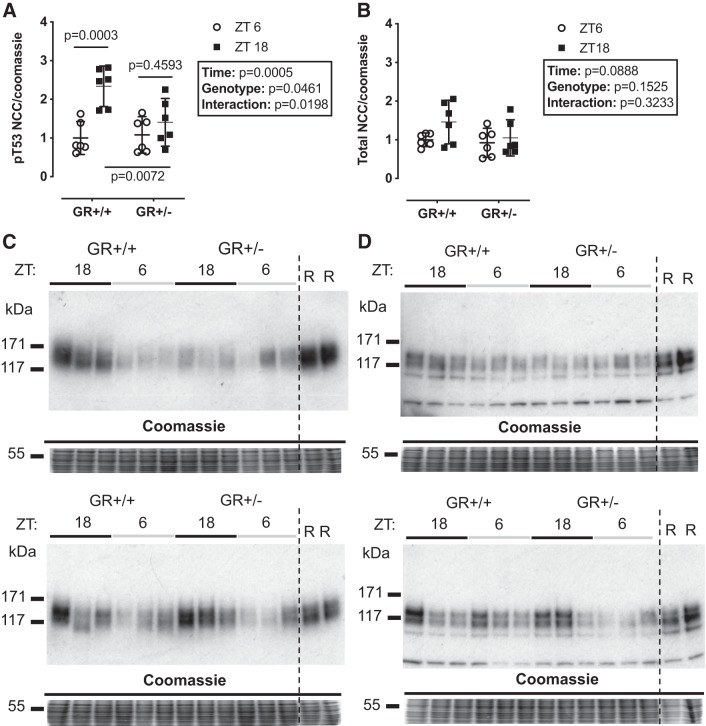
Diurnal variation in sodium-chloride cotransporter (NCC) phosphorylation (p) at Thr^53^ (T53) is blunted in animals haploinsufficient for the glucocorticoid receptor (*A*) with no changes to total protein (*B*). Kidneys from male B6N92 heterozygote mice and their littermate controls terminated at *Zeitgeber* *time* (ZT) *6* and ZT18 (lights on = ZT0, 7:00 AM local time) underwent Western blot analysis for pT53 and total protein. Western blot analyses were run in parallel (*C* and *D*). R, reference pool of kidney homogenate loaded on all gels in duplicate. Data are means ± SD and were analyzed by two-way ANOVA with post hoc Sidak’s tests, *n* = 6 biological replicates.

In a final series of experiments, we assessed diurnal pT53-NCC abundance in mice heterozygous for a null mutation in *Nr3c1*, encoding GR. Using archived protein homogenates of kidney tissue taken at ZT6 and ZT18, we found no difference in total NCC abundance between genotypes (Fig. 7*A*). However, the significant diurnal variation in pT53-NCC seen in wild-type mice was lost in GR heterozygote null animals (Fig. 7*B*).

## DISCUSSION

Our study aimed to determine the receptor pathway through which glucocorticoids regulate NCC activity and had three main findings. First, adrenal hormones influence the total pool of NCC protein available for phosphorylation via tonic activation of MR. Second, corticosterone activates the phosphorylation of NCC via GR but not MR. Third, GR activation, presumably by corticosterone, contributes to the diurnal variation of NCC phosphorylation, itself a regulator of the circadian rhythm of BP.

### 

#### MR activation regulates the total NCC pool.

Bilateral adrenalectomy or chronic spironolactone treatment of adrenal-intact mice reduced the abundance of total NCC protein, and the effects were of similar magnitude. Previous studies found that chronic spironolactone treatment reduced NCC by ~40% in adrenal-intact rats (30), and selective renal tubular deletion of MR exerts a similar effect on NCC abundance in adrenal-intact mice (50). Furthermore, aldosterone infusion in rats causes an MR-dependent increase in NCC abundance (20, 29, 52) and also enhances NCC expression in ADX animals (30, 53), although NCC abundance is not normalized (9). Similar data are reported in mice, and here the stimulatory effect of aldosterone on NCC abundance appears to be restricted to DCT2 cells (34).

The contribution of glucocorticoid/GR signaling to total NCC abundance is less clear. In our study, chronic RU486 treatment did not alter total NCC expression. In contrast to this, chronic dexamethasone increased [^3^H]metolazone binding density (reflecting NCC transporter number) and thiazide-sensitive sodium transport in vivo (reflecting transporter activity) in ADX rats (9, 53). The stimulatory effect of GR agonism was additive to aldosterone (9), suggesting that distinct receptor pathways contribute to the overall regulation of the NCC pool by adrenal steroids. However, other studies have suggested that dexamethasone, in contrast to aldosterone, cannot restore NCC expression in ADX rats (29). Overall, multiple studies in rodents have shown that aldosterone/MR signaling plays an important role in determining the amount of NCC expressed in DCT cells; the effect of glucocorticoids/GR is currently less clearly defined.

#### GR activation promotes NCC phosphorylation.

We found that NCC phosphorylation at key residues was directly affected by manipulation of in vivo glucocorticoid status (19). We now show that glucocorticoids directly increase phosphorylation of NCC at Thr^53^, used widely as a surrogate for in vivo NCC activity since it promotes membrane retention of the transporter (40, 43). Surprisingly, chronic spironolactone pretreatment did not prevent corticosterone-induced pT53-NCC, and we therefore discount glucocorticoid activation of MR. Instead, RU486 (aka mifepristone) pretreatment completely prevented phosphorylation of NCC by corticosterone. RU486 is a steroidal antagonist of GR with greater binding affinity than the endogenous glucocorticoid. It does not bind to MR but is an effective antagonist of both the progesterone receptor (6) and the androgen receptor (14). We do not consider that androgen receptor inhibition is an important factor in the current study, since previous work has shown that orchiectomy did not affect pNCC or total protein levels (16). The progesterone receptor, expressed in male kidney (36), is likely to be fully antagonized by our RU486 pretreatment protocol. Moreover, circulating progesterone in male mice originates from the adrenal gland, and adrenalectomy would therefore abolish progesterone production in our experiments. This may be relevant to our current findings. Progesterone increases NCC abundance/phosphorylation in female rodents (42) and accounts for the sexual dimorphism of elevated NCC activation in female rodents. Whether progesterone contributes significantly to NCC activation in male animals is less clear. Progesterone is typically low in male animals and also lacks a circadian rhythm (16). Overall, we cannot exclude a role for either the progesterone or androgen receptor, but the most plausible explanation of our data is that corticosterone induces phosphorylation of NCC via activation of GR.

We do not yet know the molecular mechanism connecting GR activation to NCC phosphorylation, but it is unlikely to involve SGK1; corticosterone injection increased whole kidney SGK1 abundance, which was prevented by pretreatment with either MR or GR antagonists. Plasma potassium concentration is recognized as a major regulator of NCC, with hypokalemia inducing phosphorylation of the transporter (37, 47). In patients with Cushing syndrome, phosphorylation of NCC in urinary exosomes correlated with serum potassium rather than urinary free cortisol (44). In our study, plasma potassium tended to be lower in mice receiving corticosterone, but plasma potassium was not altered by RU486 pretreatment, and we do not think this was a major factor in our study (Table 2). We did not measure plasma potassium in mice treated with spironolactone, but MR antagonism is widely recognized as inducing hyperkalemia, which in turn dephosphorylates NCC (37, 47). Mice with kidney-specific deletion of the γ-epithelial sodium channel subunit are severely hyperkalemic and show reduced NCC expression (5). Prevention of hyperkalemia through dietary potassium restriction and/or treatment with a potassium binder restored NCC abundance to control levels, suggesting that plasma potassium is a primary regulator of the NCC pool. Thus, MR blockade did not prevent phosphorylation of NCC by acute corticosterone in our experiments, but spironolactone-induced hyperkalemia could account for the observed reduction in total NCC protein.

Drawing consensus from our study and the literature, both aldosterone and corticosterone influence NCC activity via their cognate receptors. At a molecular level, it is unclear how the DCT cell discriminates between glucocorticoid/GR and aldosterone/MR signaling. Cells in DCT1 and DCT2 express both receptors (1), and expression of the glucocorticoid-metabolizing enzyme 11βHSD2 may be restricted to DCT2 or may even be absent altogether in the mouse. Thus, DCT cells are not uniformly configured as conventional “aldosterone-target” cells, and alternative processes may be in play. It is possible, for example, that MR can discriminate between aldosterone and glucocorticoid in the absence of 11βHSD2 (22). Recent research has shown that G protein-coupled receptor 30, the membrane receptor accounting for the rapid nongenomic actions of estrogen (35), is also important for rapid plasma potassium-dependent activation of NCC by aldosterone (10). Certainly, pathways of adrenal steroid signaling in the DCT are not resolved and cannot be explained by classical definitions.

#### The GR in the diurnal rhythm of pNCC.

We confirmed the diurnal rhythm of pNCC (19, 49), further finding that chronic systemic GR antagonism perturbed this rhythm. This prompted assessment of diurnal variation in pNCC in mice with a genetically induced reduction in GR expression (18, 27). We were only able to assess pNCC in GR heterozygote null mice at two time points using archived kidney protein homogenates, the midpoints of the subjective day and night, but our data indicate that reduced GR expression significantly dampened the pNCC phosphorylation rhythm. This complements our previous work in which we introduced arrhythmicity of plasma corticosterone, either by adrenalectomy or by pharmacological clamping in the midphysiological range (19). Overall, these two studies show that impairment of dynamic GR signaling suppresses pNCC rhythmicity. The effect of MR blockade was not assessed here because our previous work indicated that aldosterone was not a major regulator of pNCC rhythmicity (19). Nevertheless, others have shown that chronic eplerenone treatment dampened pNCC rhythm (49), and we cannot therefore exclude a role of aldosterone/MR signaling.

The molecular mechanisms through which GR contributes to pNCC rhythm are not clear. It is probable that GR engages with the DCT clock since glucocorticoids have a major role in synchronizing peripheral clocks to the master clock in the SCN (4, 33). It is also probable that the DCT clock integrates with NCC regulatory pathways at some level, since Per1 and Bmal null mice demonstrate altered NCC expression (13, 51). Moreover, clock genes may regulate NCC by acting as transcription factors to genes within the NCC regulation pathways. Indeed, both WNK4 and WNK1 contain E-box-binding sites, interaction sites for Per1 and Cry1 (39). Blocking the nuclear entry of Per1 in vitro decreased NCC activity through transcriptional regulation of NCC, WNK1, KS-WNK1, and WNK4 (39). We therefore profiled clock gene expression in our glucocorticoid manipulation experiments. In ADX mice, corticosterone robustly increased the expression of Per1, Per2, Cry1, and Bmal. In adrenal-intact mice, corticosterone increased only Per1 and Per2 expression 4 h posttreatment, and this effect was blocked by either MR or GR inhibition. When assessed at 6-h intervals, all clock genes displayed robust rhythms, and, surprisingly, these were not greatly altered by GR blockade. One interpretation is that kidney clocks are intrinsic and autonomous; whereas they can be entrained by GC stimulation, they do not require stimulation to cycle. Consistent with this idea, clock protein rhythms are maintained in ADX rats (48), and liver-specific GR ablation did not alter baseline clock rhythms in the liver (21). Chronic RU486 abolished the pNCC rhythm, suggesting that this operates independent of the DCT clock. Tubule-specific Bmal knockout mice have no sodium-handling defects, suggesting that the tubular rhythms may be imposed by external cues (31). An alternative explanation is that even small changes in Per1 rhythm affect the pNCC rhythm; in distal nephron-specific Per1 knockout mice, NCC expression was increased (13), and Per1 has been shown to interact with NCC-regulatory pathways (39). It is difficult from our experiments to draw strong conclusions about clock operation in the DCT. Measurement of mRNA expression at the whole kidney level lacks refinement, and microarray experiments performed on isolated segments of DCT/connecting tubule and cortical collecting duct show clear segmental differences in both the number of genes that oscillate over a 24-h period and in the temporal distribution of the acrophases of those genes (54). This is an important limitation of our study, and operation of the DCT clock in vivo remains mysterious.

In conclusion, our study demonstrates that glucocorticoids, acting via GR, are regulators of NCC activity and contribute to defining activity over the 24-h cycle. Because NCC contributes importantly to sodium and potassium homeostasis, and BP control, our findings are relevant to the increased cardiovascular risk associated with human conditions of glucocorticoid excess or impaired diurnal rhythmicity in circulating cortisol.

## GRANTS

This work was funded by an Intermediate Fellowship (IN001-2018) and project grant (RP39/2011) from Kidney Research UK and PhD studentships from The British Heart Foundation (FS/11/78/29328, FS/15/63/32033, and FS/16/54/32730).

## DISCLOSURES

No conflicts of interest, financial or otherwise, are declared by the authors.

## AUTHOR CONTRIBUTIONS

J.R.I. and M.A.B. conceived and designed research; J.R.I., N.K.J., H.M.C., M.K.M., and T.S.P. performed experiments; J.R.I., N.K.J., H.M.C., M.K.M., and T.S.P. analyzed data; J.R.I., N.K.J., H.M.C., and M.A.B. interpreted results of experiments; J.R.I., N.K.J., H.M.C., and M.K.M. prepared figures; J.R.I. and M.A.B. drafted manuscript; J.R.I., P.W.F., and M.A.B. edited and revised manuscript; J.R.I., N.K.J., H.M.C., P.W.F., and M.A.B. approved final version of manuscript.
